# Supplementary material for: A fault-intrusion-tolerant system and deadline-aware algorithm for scheduling scientific workflow in the cloud
Source: PeerJ Comput Sci. 2021 Nov 2;7:e747. doi: 10.7717/peerj-cs.747 (PMC8576554; doi:10.7717/peerj-cs.747)
Supplement: Supplemental Information 6 [file peerj-cs-07-747-s006.zip › Myproject1.1/docs/index-files/index-10.html]

J-Index


JavaScript is disabled on your browser.


- Overview
- Package
- Class
- Use
- Tree
- Deprecated
- Index
- Help

- Prev Letter
- Next Letter

- Frames
- No Frames

- All Classes

A B C D E F G H I J L M N O P Q R S T U V W Z 


## J

Job - Class in org.workflowsim
:   Job is an extention to Task.

Job(int, long) - Constructor for class org.workflowsim.Job
:   Allocates a new Job object.

JOB\_SUBMIT - Static variable in class org.workflowsim.WorkflowSimTags


jobId - Variable in class org.workflowsim.failure.FailureRecord
:   the job id.

jobid2record - Static variable in class org.workflowsim.failure.FailureMonitor
:   JobID to a Failure Record.

jobList - Variable in class org.workflowsim.ClusteringEngine
:   The job list

jobsList - Variable in class org.workflowsim.WorkflowEngine
:   The job list.

jobsReceivedList - Variable in class org.workflowsim.WorkflowEngine
:   The job received list.

jobsSubmitted - Variable in class org.workflowsim.WorkflowEngine
:   The job submitted.

jobsSubmittedList - Variable in class org.workflowsim.WorkflowEngine
:   The job submitted list.

A B C D E F G H I J L M N O P Q R S T U V W Z

- Overview
- Package
- Class
- Use
- Tree
- Deprecated
- Index
- Help

- Prev Letter
- Next Letter

- Frames
- No Frames

- All Classes
